# Biotinylated Cyclooligosaccharides for Paclitaxel Solubilization

**DOI:** 10.3390/molecules23010090

**Published:** 2018-01-02

**Authors:** Eunae Cho, Seunho Jung

**Affiliations:** 1 Institute for Ubiquitous Information Technology and Applications (UBITA) & Center for Biotechnology Research in UBITA (CBRU), Konkuk University, 120 Neungdong-ro, Gwangjin-gu, Seoul 05029, Korea; echo@konkuk.ac.kr; 2 Department of Bioscience and Biotechnology, Microbial Carbohydrate Resource Bank (MBRC) & Center for Biotechnology Research in UBITA (CBRU), Konkuk University, 120 Neungdong-ro, Gwangjin-gu, Seoul 05029, Korea

**Keywords:** solubilization, cyclooligosaccharide, paclitaxel, biotin

## Abstract

The poor water solubility of paclitaxel causes significant problems in producing cancer therapeutic formulations. Here, we aimed to solubilize paclitaxel using biocompatible cyclic carbohydrates. Generally recognized as safe, labeled β-cyclodextrin (β-CD), a cyclic α-1,4-glucan consisting of seven glucoses, was prepared, and bio-sourced cyclosophoraoses (CyS), which are unbranched cyclic β-1,2-glucans with 17–23 glucose units, were purified using various chromatographic methods from *Rhizobium leguminosarum* cultural broth. For effective targeting, CyS and β-CD were modified with a biotinyl moiety in a reaction of mono-6-amino CyS and mono-6-amino-β-CD with *N*-hydroxysuccinimide ester of biotinamidohexanoic acid. Interestingly, the aqueous solubility of paclitaxel was enhanced 10.3- and 3.7-fold in the presence of biotinyl CyS and biotinyl β-CD, respectively. These findings suggest that biotin-appended cyclooligosaccharides can be applied to improve the delivery of paclitaxel.

## 1. Introduction

Paclitaxel (PTX) is a complex diterpene natural product isolated from the stem bark of *Taxus brevifolia* [1]. Because it induces cell death by binding to β-tubulin and affecting microtubule dynamics [2], PTX has shown excellent antitumor activity for treating metastatic breast, advanced ovarian, and non-small cell lung cancer [3]. However, its clinical usage has been limited by its poor water solubility, making the development of injectable formulations difficult [4]. Poor aqueous solubility is also a serious problem associated with drug discovery and combinatorial screening systems, and at least 40% of identified active substances are poorly soluble in water [5]. To increase the water solubility of hydrophobic drugs, nanosizing, liposomes, and synthesized water-soluble derivatives have been utilized [6,7,8]. Although co-solvent systems including ethanol and Cremophore have also been employed, significant side-effects such as neurotoxicity, nephrotoxicity, and anaphylactoid hypersensitivity reactions occur [9]. Therefore, there is a growing need for alternative biocompatible strategies to solubilize hydrophobic drugs.

For drug solubilization, supramolecular complexation technology has been introduced and the recognition ability of carbohydrates is attractive for intermolecular interactions [10]. Supramolecular complexation with appropriate host molecules can solubilize hydrophobic guest molecules, separate chiral compounds, and catalyze organic transformation. Particularly, macrocyclic carbohydrate hosts, such as cyclodextrins (CD) and cyclosophoraose (CyS), have received attention because of their characteristic structural features, including hydrophobic cavities, hydrophilic exteriors, and selective molecular recognition abilities [11,12]. CDs are α-1,4-linked glucans composed of 6, 7, or 8 glucose units for α-, β-, and γ-CD, and are produced by cyclodextrin glycosyl transferase treatment of starch. Using β-cyclodextrin (β-CD) as a complexing agent, the stability and solubility of various drugs containing ibuprofen, itraconazole, and atenolol have been enhanced [13,14,15]. With the availability of various guest molecules due to the large size and different glycosidic linkages, CyS are produced via the metabolic processes of many fast-growing soil bacteria such as *Rhizobium* species in their periplasmic space as intra- and extra-oligosaccharides [16,17]. CyS, which are cyclic β-1,2 glucans with 17–23 glucose residues, have been reported to exhibit induced fit complexation with hydrophobic bioactive molecules [12,18,19].

To endow cyclooligosaccharides with advanced functionality, they can be modified with various substrates including methyl, carboxy-methyl, and hydroxypropyl groups [20]. Additionally, original β-CD shows a limitation: a rigid and stable cavity size (7 Å) [21]. When breaking ordered hydrogen bonds of secondary hydroxyl groups, they show increased water solubility and expanded guest ranges [22]. At the point of site-specific delivery, specific CD derivatives, including biphenylylacetic acid or methicilline-β-CD conjugates, have been reported [23,24]. Here, we modified cyclooligosaccharides with biotin, whose receptor is overexpressed on the surface of cancer cells [25], to achieve cancer cell specificity. Biotin is a growth promoter known as vitamin B_7_ and is composed of a valeric acid and ureido ring fused with a tetrahydrothiophenene ring. Although the paclitaxel solubilization is important for its clinical effectiveness in anticancer therapy, and thus researchers have consistently studied the topic so far [26,27,28], more advanced paclitaxel delivery systems are still challengeable [29]. Here, our own strategy is the use of cell-derived cyclooligosaccharides and their biotinyl derivatives.

In the present study, we isolated and purified bio-sourced CyS using ethanol precipitation and chromatography. The mono-biotinylation of the primary hydroxyl groups of cyclooligosacchrides was successfully performed through a mono-tosylation, azidation, amination, and 1-ethyl-3-(3-dimethylaminopropyl) carbodiimide (EDC) coupling reaction, and the resulting structure was analyzed by nuclear magnetic resonance (NMR) spectroscopy and matrix-assisted laser desorption/ionization (MALDI) time-of-flight (TOF) mass spectrometry. Based on the flexible and distorted rings of various sizes, CyS and biotinyl derivatives were evaluated for their ability to solubilize paclitaxel in a phase solubility study, and the representative cyclic host β-CD and derivatives were also compared.

## 2. Results and Discussion

### 2.1. Synthesis and Structural Analyses of Biotinyl Cyclooligosaccharides

**Scheme 1:**
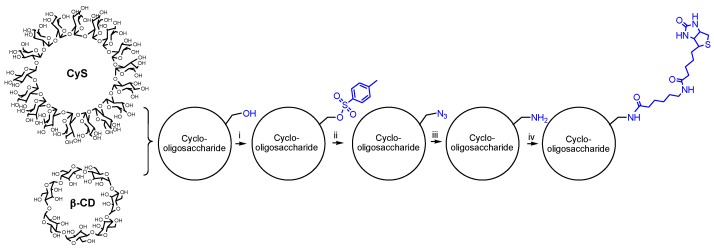
Synthetic scheme for biotinyl cyclooligosaccharides. (i) Monotosylation step; (ii) azidation step; (iii) amination step; and (iv) biotinylation step.

**Figure 1:**
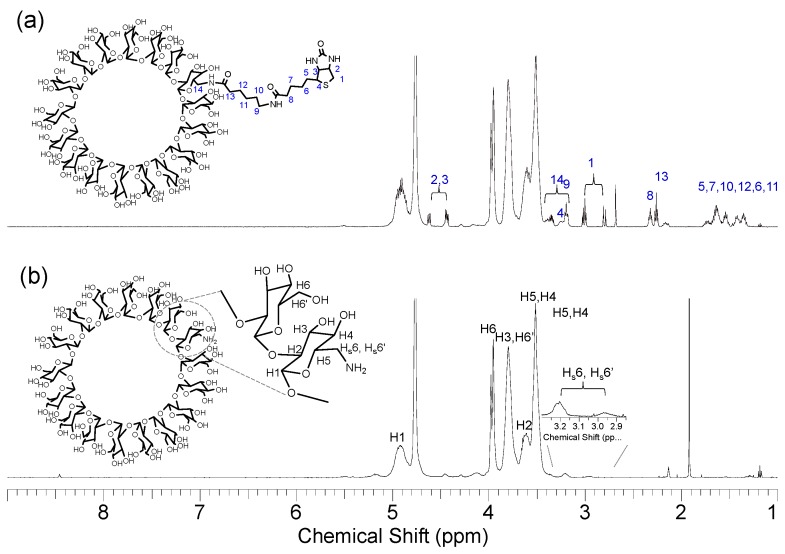
^1^H NMR spectra of (**a**) biotinyl cyclosophoraose (CyS); and (**b**) amino CyS. Inset shows the corresponding chemical structures (NMR solvent: D_2_O).

**Figure 2:**
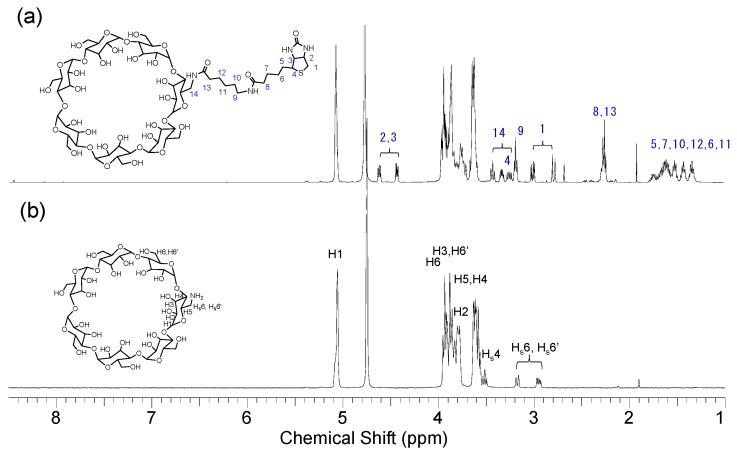
^1^H NMR spectra of (**a**) biotinyl β-cyclodextrin (β-CD); and (**b**) amino β-CD. Inset shows the corresponding chemical structures (NMR solvent: D_2_O).

Rhizobial CyS, which is a cyclic beta 1,2 glucan with 17–23 glucose units, was purified using chromatographic methods and the structure was confirmed by MALDI-TOF mass spectrometry, NMR analysis, and Fourier-transform infrared spectroscopy [19]. From the initial unbranched cyclic structure, mono-6-amino CyS was prepared via monotosylation, azidation, and amination, as described in the Materials and Methods (Scheme 1). Next, in addition to the peaks between 3.40 and 5.10 ppm assigned to the H1–H6 protons of glucose units, signals for H_s_6 and H_s_6’ protons with an amino group appeared at 0.77 and 0.83 ppm lower in the field than those of H6 and H6’ of unsubstituted glucoses (Figure 1). The mono-6-amino CyS was tethered by biotin with an amidocaproyl linker in an EDC coupling reaction with biotinamidohexanoic acid *N*-hydroxysuccinimide ester. The characteristic H_s_6 peaks at 3.21 and 2.97 ppm disappeared, and 14 proton signals appeared at 3.35 and 3.21 ppm because of the next amide bond. The presence of the ureido and tetrahydrothiophenene-fused ring caused proton peaks (1–4) at approximately 4.40–4.63, 3.26, and 2.70–3.03 ppm. Other 5–13 proton peaks were observed from aliphatic chains from the amidocaproyl linker and valeic acid part. In the same manner, mono-6-amino-β-CD was prepared, and identical H_s_6 peaks appeared ranging from 2.90 to 3.20 ppm as a ddd signal due to an AB pattern (Figure 2) [30]. H_s_4 protons were also clearly observed in the upfield shifted region compared with original H4 protons of glucoses. After biotinyl functionalization, the patterns of other signals were similar to that of biotinyl CyS. 

**Figure 3:**
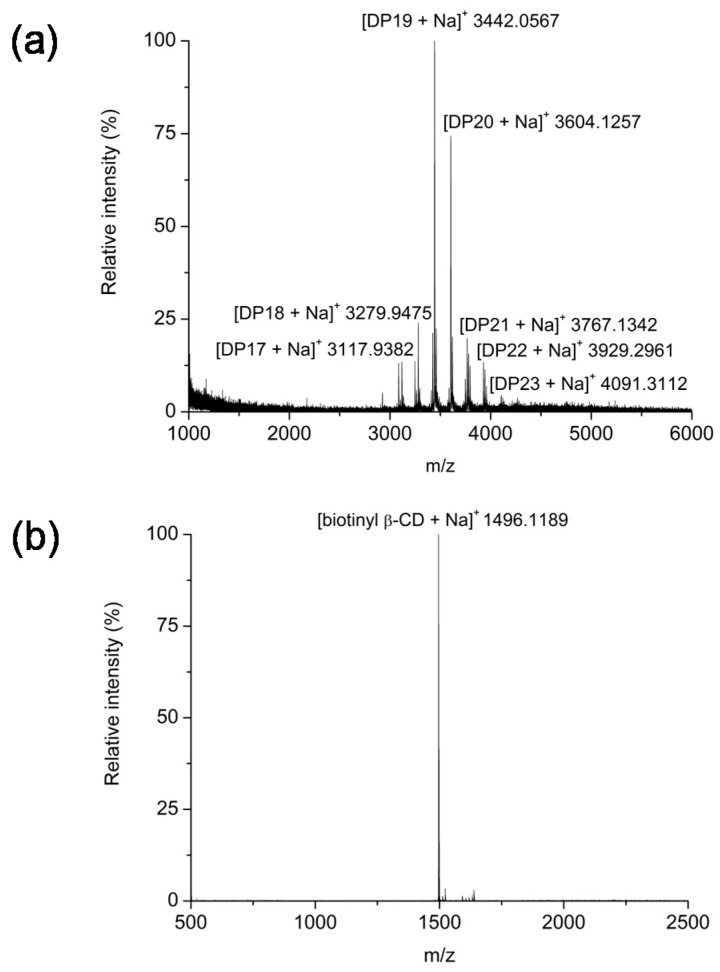
MALDI-TOF mass spectra of biotinyl cyclooligosaccharides. (**a**) Biotinyl CyS; and (**b**) biotinyl β-CD.

The molecular weights (MW) of synthesized biotinyl CyS and biotinyl β-CD were determined by mass spectrometry (Figure 3). Because CyS has a degree of polymerization (DP) from 17 to 23, the biotinyl CyS showed increased masses according to the respective DP. The molecular ions were assigned as [DP17 + Na]^+^, [DP18 + Na]^+^, [DP19 + Na]^+^, [DP20 + Na]^+^, [DP21 + Na]^+^, [DP22 + Na]^+^, and [DP23 + Na]^+^, with signals at *m*/*z* 3117.9832, 3279.9475, 3442.0567, 3604.1257, 3767.1342, 3929.2961, and 4091.3112, respectively. For the biotinyl β-CD, the sodium adduct peak of the product was observed at *m*/*z* 1496.1189, which is differentiated from the molecular weight (1134.9842) of β-CD consisting of seven glucose residues. These results indicate that biotin tethered macrocyclic host molecules were successfully synthesized. In addition, because of the amidocaproyl linker between biotin and cyclooligosaccharide, the possibility of a self-inclusion state can also be avoided, and an efficient supramolecular interaction with other compounds can be expected. Since monosubstituted β-cyclodextrin can undergo self-inclusion [31], the reduction of competitive interaction using a long linker is favorable for a target molecule.

### 2.2. Biocompatibility of Cyclooligosaccharides and the Biotinyl Derivatives

**Figure 4:**
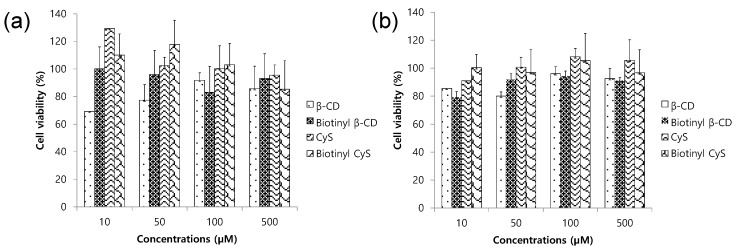
Cell viability after exposure to cyclooligosaccharides and biotinyl derivatives. (**a**) HEK 293 cells; and (**b**) HeLa cells.

To assess the biocompatibility of the prepared cyclooligosaccharides and biotinyl derivatives, a water soluble tetrazolium salts (WST) assay, which is a useful method for quantifying cytotoxicity [32], was conducted using the carbohydrate host molecules. Figure 4 shows the effects of four types of macrocyclic hosts on HEK 293 cells and HeLa cells. The normal cell viabilities compared to those of untreated cells were β-CD: 85.9%, biotinyl β-CD: 93.0%, CyS: 95.6%, and biotinyl CyS: 85.3% at 500 µM. Furthermore, cancer cell viability was more than 90% in the presence of four compounds (β-CD: 92.6%, biotinyl β-CD: 91.1%, CyS: 105.7%, and biotinyl CyS: 96.7%). Compared with the results for generally recognized as safe-labeled β-CD (GRN 000074 in October 2001) [33], no significant decreases were observed in all samples. Because cell viability less than 70% is regarded as toxic [34], materials such as β-CD, biotinyl β-CD, CyS, and biotinyl CyS are considered non-toxic to normal and cancer cell lines. This suggests that the cyclooligosaccharides and biotinyl derivatives can be used as biocompatible functional materials for future biomedical applications.

### 2.3. Phase Solubility Study

**Figure 5:**
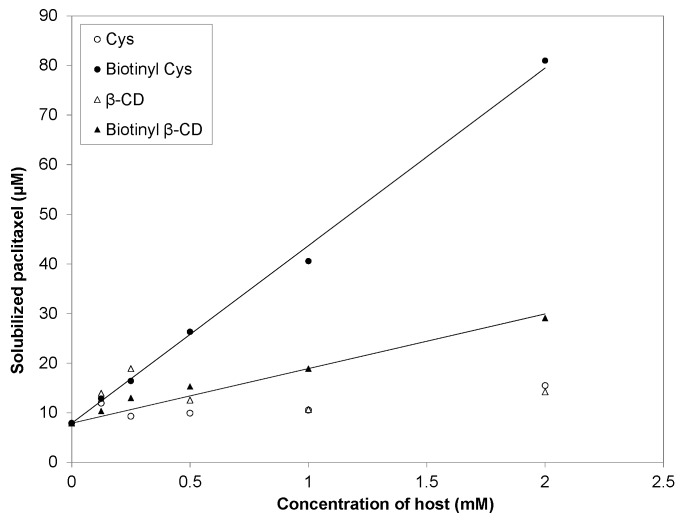
Phase solubility diagram.

Here, the effect of macrocyclic carbohydrate molecules on the solubilization of anticancer paclitaxel was investigated. In supramolecular chemistry, the phase solubility technique is generally used to evaluate the effect of host molecules on drug solubility [35]. The UV absorption of hydrophobic paclitaxel in water noticeably and gradually increased as the concentration of biotinyl CyS increased (Figure 5). The linear correlation forming an A_L_-type curve indicated a 1:1 association of paclitaxel and biotinyl CyS [36], and the association constant (K_a_) was found to be 4703 M^−1^. Solubilizing efficiency is defined as the ratio between drug solubility in the presence of a host molecule at a certain concentration and drug intrinsic solubility in water. The solubilizing efficiency of biotinyl CyS of 2 mM was 10.3, indicating a 10.3-fold increase in solubility, which is a much higher value than those of other cyclooligosaccharides.

Biotinyl β-CD also showed a linear plot for the phase solubility diagram and the solubilization effect on paclitaxel was relatively low compared with that of biotinyl CyS. The association constant of paclitaxel and biotinyl β-CD was 1409 M^−1^ and the solubilizing efficiency was 3.7. The result is reasonable if the flexible and large-sized ring structure of CyS is more favorable than β-CD containing a small cavity and rigid hole, in the case of the supramolecular association with paclitaxel. Molecular recognition of substrates by CyS is attributed to the noncovalent interactions of their hydrophobic entities or hydroxyl groups in CyS [37]. CyS is microbial cyclic polysaccharide with β-1-2 glucans, which has both better aqueous solubility and more diverse flexible hydrophobic cavities compared with β-CD [38]. The superior efficiency of CyS may be because of differences in physicochemical and structural properties compared with those of the CD analogs. In addition, biotin and amidocaproyl moieties positively affect the solubilization of paclitaxel, which could be because of the effective noncovalent interaction with paclitaxel due to the additional hydrogen bonding sites between the amide bond in biotinyl cyclooligosaccharides and ester linkages in paclitaxel. The initial increase and decrease pattern of paclitaxel solubility by β-CD may be related to the partial complexation between these molecules. Although the aromatic rings of paclitaxel can be incorporated in the cavity of β-CD, considering the cavity size (7 Å) and benzene ring [21], the complexed structure is not very soluble in water because of its other bulky moiety. In actual fact, the extrapolate area of paclitaxel is 31.74 Å^2^ [39]. CyS may have a collapsed structure because of some intramolecular associations [40] and the interaction with paclitaxel may be limited. In addition, the small cavity size of 3.7 Å and the non-symmetric conformation inferred from NMR spectroscopic and a molecular modeling study, support this [17]. These results demonstrate that efficient supramolecular interactions between biotinyl CyS and paclitaxel contribute to increasing paclitaxel solubility in water. Thus, the hydrophobic aggregation of paclitaxel in water may be noticeably reduced because of the association with biotinyl CyS and dispersion by biotinyl CyS.

### 2.4. ^1^H NMR Analysis

**Figure 6:**
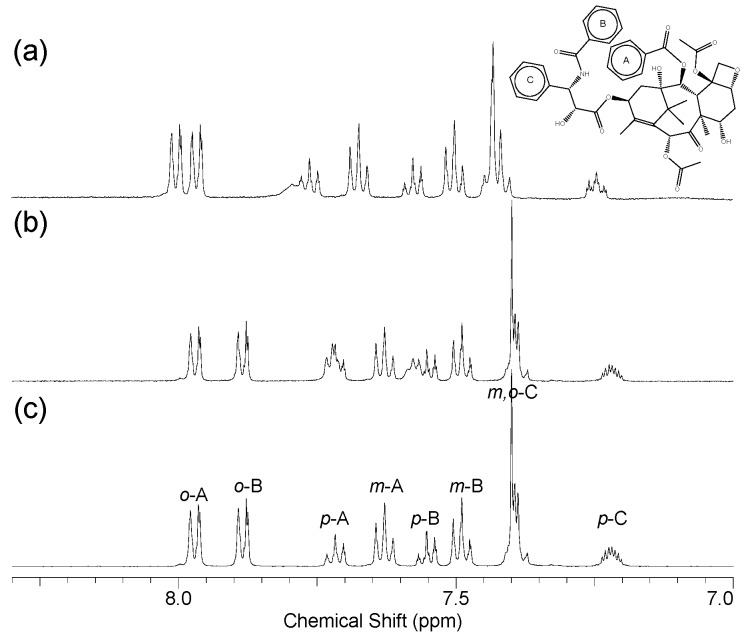
^1^H NMR spectra. (**a**) Paclitaxel interacted with biotinyl CyS; (**b**) Paclitaxel interacted with biotinyl β-CD; and (**c**) Paclitaxel alone (NMR solvent: DMSO-*d*_6_, 99%). Inset shows the chemical structure of paclitaxel.

To confirm and compare the interaction between paclitaxel and biotinyl CyS, ^1^H NMR spectroscopy was performed. ^1^NMR spectroscopy reveals valuable information about noncovalent interactions at the molecular level [41]. Noncovalent interactions are indicated as protons shifted upfield or downfield by the molecular environmental change. Figure 6 shows the noticeable chemical shift in the aromatic ring of paclitaxel by biotinyl CyS, with the values summarized in Table 1. In particular, protons of the A, B, and C aromatic rings were affected, indicating a local polarity change in the moiety in the presence of biotinyl CyS. The splitting pattern of C aromatic ring protons was also clearly altered after complexation with biotinyl CyS, with the largest chemical shift change observed in *ortho* protons of the B aromatic ring. A new resonance at 7.80 ppm appeared next to the *para* proton of the A aromatic ring. Although biotinyl β-CD was relatively less affected by the proton resonance of paclitaxel, the splitting pattern of *para* protons of the A aromatic ring changed, and new peaks near the *para* protons of B aromatic ring were observed. Thus, some interaction is suspected, which is correlated with the small effect (2.8-fold) on the paclitaxel solubility enhancement by some association with paclitaxel as shown in Figure 5. This molecular leveled evidence from NMR spectroscopic analysis confirms the efficient supramolecular interaction between paclitaxel and biotinyl CyS as a key factor affecting the solubility increase of paclitaxel.

## 3. Materials and Methods

### 3.1. Materials

β-CD was obtained from Sigma-Aldrich Chemical Co. (St. Louis, MO, USA). Paclitaxel was provided by Samyang Genex Corporation (Seoul, Korea). Sodium azide, 1-(*p*-toluenesulfonyl) imidazole, and *p*-toluenesulfonyl chloride were purchased from Tokyo Chemical Industry (Tokyo, Japan). Triphenylphosphine (99%) and biotinamidohexanoic acid *N-*hydroxysuccinimide ester were purchased from Sigma Aldrich (St. Louis, MO, USA). *N*,*N*-Dimethylformamide (anhydrous, 99.8%) was obtained from Alfa Aesar (Haverhill, MA, USA), and acetone was obtained from Avantor. All other chemicals used in the study were of analytical grade.

### 3.2. Isolation and Purification of CyS

*Rhizobium leguminosarum* VF-39 was provided by the Microbial Carbohydrate Resource Bank, Konkuk University, Korea. Cells were grown in a GMS medium at 25 °C for 14 days. CyS was refined from *R. leguminosarum* VF-39, as described previously [42]. After removing the cells, the culture supernatants were concentrated by five-fold. After adding two volumes of ethanol, the alcoholic supernatant fluid was concentrated, and the concentrate was re-precipitated by adding 7 volumes of ethanol. The precipitate was applied on a Bio-Gel P6 column, and the fractions containing desired glucans were concentrated and desalted. Purified CyS was characterized by NMR spectroscopic and MALDI-TOF mass spectrometric analyses.

### 3.3. Synthesis of Biotinyl Cyclooligosaccharides

Biotin tethering to cyclooligosaccharides starts from monotosylation on the primary hydroxyl group, but the monotosylation method of both cyclooligosaccharides slightly differed from each other. For monotosylation of β-CD, β-CD (10.0 g, 8.8 mmol) was dissolved in water (250 mL) and heated to 60 °C. After cooling to room temperature, 1-(*p*-toluenesulfonyl) imidazole (6 g, 27 mmol) was added and mixed for 6 h. Sodium hydroxide (4.5 g, 112.5 mmol) solution in water (12.5 mL) was added dropwise for 20 min. After filtration, the filtrate was quenched using ammonium chloride (12.05 g, 225 mmol), and the resulting mixture was subjected to air flow for drying. The precipitated product was washed with water and acetone. The yield of monotosylated β-CD was 28%. For monotosylated CyS, CyS (1 g, 0.28 mmol) was dissolved in water (50 mL), and copper sulfate (1 g, 4 mmol) in water (100 mL), was added. NaOH (1 g, 25 mmol) solution in water (50 mL) was mixed and stirred for 10 min. The suspension became dark blue, and *p*-toluenesulfonyl chloride (2.5 g, 13.2 mmol) dissolved in 0.1 mL of acetonitrile was added in a dropwise manner for 1 h. The suspension was stirred at room temperature for 4 h then neutralized with ammonium chloride. After removing the precipitate, the filtrate was concentrated and desalted on a Bio-Gel P2 column. The sample was subjected to semi-preparative HPLC (Agilent Technologies 1200 series, Santa Clara, CA, USA) on a reverse-phase column (Eclipse XDB-C18, 9.4 × 250 mm, 5 µm) at room temperature. The yield of monotosylated CyS was 25%. Further azidation and amination were performed, as described in our previous report [43,44]. The mono-6-amino-β-CD or mono-6-amino-CyS were dissolved in DMF and reacted with biotinamidohexanoic acid *N*-hydroxysuccinimide ester for 24 h at 40 °C. After acetone precipitation, the samples were desalted and lyophilized. The overall synthetic yield from original cyclooligosaccharide to biotinylated one is about 8%. The structures of the resulting biotinyl cyclooligosaccharides were analyzed by NMR spectroscopy and MALDI-TOF mass spectrometry.

### 3.4. MALDI-TOF MS and NMR Spectroscopic Analysis

NMR spectroscopic analysis was performed on a Bruker (AMX, Billerica, MA, USA) spectrometer (500 MHz) with deuterium water (D_2_O; 99.9 at. % D or DMSO-*d*_6_). All NMR measurements were performed with 0.6 mL samples in 5 mm NMR tubes. MALDI-TOF experiments were essentially performed using a Voyager-DETM STR BioSpectrometry (PerSeptive Biosystems, Framingham, MA, USA) in positive ion reflector mode. Samples were prepared according to the dried droplet method by using 2,5-dihydroxybenzoic acid as a matrix.

### 3.5. Cytotoxicity Assay 

The cytotoxicity of cyclooligosaccharides and biotinyl derivatives was assessed by a WST assay on HEK293 and HeLa cells [45]. The assays were performed by incubating 5 × 10^3^ cells/well with increasing concentrations (0, 10, 50, 100, and 500 µM) in a 96-well plate (Corning Costar, Corning, NY, USA) at 37 °C in a humidified incubator containing 5% carbon dioxide for 24 h. After treatment of cyclooligosaccharides between 0 and 500 μM, the cells were incubated for 24 h. Next, 10 µL of WST-1 was added to each well, and the cells were incubated at 37 °C for 4 h. The absorbance of each well at 450 nm was measured using a SpectraMax 190 microplate reader (Molecular Devices Corporation, Sunnyvale, CA, USA). Cell viability was calculated as follows.

V (%) = A/A_0_ × 100 (1)

where A and A_0_ are the absorbance values after and before addition of cyclooligosaccharides, respectively. Experiments were performed in triplicate.

### 3.6. Phase Solubility Studies

Paclitaxel (2 mM) was dissolved in methanol and this solution (0.25 mL) was added to aqueous solutions containing β-CD, biotinyl β-CD, CyS, and biotinyl CyS (0.0–4.0 mM), in vials. The mixed solution was stirred at 26 °C for 24 h then the methanol was evaporated using nitrogen gas. After lyophilization, the samples were dissolved in water (0.5 mL) and the precipitate was removed by centrifugation. After dilution, the solubilized paclitaxel was analyzed at 260 nm using a UV visible spectrometer (UV 2450, Shimadzu Corp., Kyoto, Japan) [46]. The apparent association constants (K_a_) of paclitaxel/cyclooligosaccharides were calculated from the phase solubility diagram according to Higuchi and Connor’s method [35].

K_a_ (M^−1^) = slope/S_i_(1-slope) (2)

where S_i_ is the initial solubility of paclitaxel equilibrated in water.

## 4. Conclusions

The poor water solubility of paclitaxel limits its clinical usage because of injection difficulties. To overcome this problem, supramolecular complexation technology using carbohydrate host molecules was attempted. In the present study, bio-sourced CyS, cyclic β-1,2 glucans with DP 17–23, was purified and modified with a biotin group via mono-tosylation, azidation, amination, and EDC coupling reaction. GRAS β-CD, a cyclic α-1,4-glucan with DP 7, was also prepared and tethered with a biotin group in the same manner. The results of the WST cytotoxicity assay on normal and cancer cell lines suggested that the modified and original macrocyclic carbohydrates can be used as drug solubilizers to improve biocompatibility. Accordingly, using those molecules, the effect on paclitaxel solubility in water was investigated. In particular, biotinyl CyS and β-CD showed solubilization efficiencies of 10.3 and 3.7, with apparent association constants (K_a_) of 4703 and 1409 M^−1^, respectively. The supramolecular interaction between paclitaxel and biotinyl cyclooligosaccharides was also determined at the molecular level by NMR spectroscopy. The present results may be important for biomedical applications, to resolve solubility problems and target oriented advantages as novel strategies for anticancer drug delivery.

## Figures and Tables

**Table 1:** Chemical shift changes in aromatic rings of paclitaxel in the presence of biotinyl CyS and biotinyl β-CD.

	Paclitaxel	Biotinyl CyS (ppm)		Biotinyl β-CD (ppm)	
Protons	δ ^b^	δ ^a^	Δδ	δ ^a^	Δδ
*o*-A	7.97	8.00	0.03	7.97	0
*m*-A	7.63	7.67	0.04	7.63	0
*p*-A	7.72	7.76 (7.80) ^c^	0.04	7.72 ^d^	0
*o*-B	7.88	7.96	0.08	7.88	0
*m*-B	7.49	7.50	0.01	7.49	0
*p*-B	7.55	7.58	0.03	7.55 (7.57) ^c^	0
*o*-C	7.39	7.42 ^d^	0.03	7.39	0
*m-C*	7.40	7.43 ^d^	0.03	7.40	0
*p-C*	7.22	7.25 ^d^	0.03	7.22	0

δ ^a^ = after complexation with biotinyl cyclooligosaccharides; δ ^b^ = before complexation with biotinyl cyclooligosaccharides; ^c^ new peak; ^d^ changed splitting pattern. Δδ = δ_a_ − δ_b_.
